# Proteolytic enzyme-treated seaweed co-product (*Ulva laetevirens*) inclusion in corn-soybean and European broiler diets to improve digestibility, health, and performance

**DOI:** 10.1016/j.psj.2022.101830

**Published:** 2022-03-07

**Authors:** L. Stokvis, R.P. Kwakkel, W.H. Hendriks, J. Kals

**Affiliations:** ⁎ Wageningen University & Research, Wageningen Livestock Research, Wageningen 6708 WD, the Netherlands; † Wageningen University & Research, Animal Nutrition Group, Wageningen 6708 WD, the Netherlands

**Keywords:** seaweed, broiler nutrition, novel feed source, digestibility, enzyme

## Abstract

The impact of addition of an *Ulva laetevirens* (previously *Ulva rigida*) co-product treated with a broad-spectrum endo-protease when added to a standard corn-soy (**S**) based diet and a diet based on European protein sources (**EU**) on performance, in vivo digestibility and multiple gastrointestinal characteristics was investigated in broilers. In total, 624 Ross 308 one-day-old male broilers were fed one of 6 experimental diets (8 replicates) consisting of a basal diet (S or EU), or a basal diet including the *U. laetevirens* co-product (**U**) treated without (**U-**) or with (**U+**) a proteolytic enzyme. Starter diets contained 0 (wk 1) and 2.5 (wk 2), and the grower diets (wk 3 and 4) 5% seaweed co-product. In the last 2 wk, birds fed the S vs. EU grower diets showed a higher BW, BWG, and FI, as well as a lower FCR (−0.05 g/g) in wk 3 (*P* < 0.05). Heavier gizzards (+13%; *P* < 0.001) and heavier gizzard contents (+92%; *P* < 0.001) were observed in birds fed the EU vs. S diets, as well as longer villi (+8%; *P* = 0.010). U diets had a higher water holding capacity than the basal diets (+19%). In wk 4, U inclusion resulted in increased FCR (+0.06 g/g; *P* < 0.001), water intake (+7%; *P* < 0.001), and duodenal cross section (+5%; *P* = 0.033). Enzyme treatment did not affect digestibility of any nutrients, except for ash which was increased in birds fed U+ vs. U- diets (+60%; *P* < 0.001). U in S diets led to higher, and U in EU diets led to lower apparent pre-cecal digestibility of all nutrients (*P* < 0.001 for all nutrients). Although for both diet types performance was decreased, dietary *U. laetevirens* inclusion had different effects when added to a standard corn-soy diet and a diet based on European protein sources. No obvious health effects were observed, leading to the conclusion of the absence of performance of health promoting bioactive components in the *U. laetevirens* co-product, or of diminishing of these effects due to the proteolytic enzyme treatment.

## INTRODUCTION

Novel and existing feed ingredients for broiler diets are routinely investigated to create and sustain a future-proof poultry production. The seaweed *Ulva laetevirens* might contribute to the latter goal as arable land or fresh water are not needed for their production and the nutritional composition is potentially favorable, as *U. laetevirens* can have a protein content of up to 38% on a **DM** basis (Biancarosa et al., 2017; Øverland et al., 2019). In addition, health-promoting properties have been attributed to *Ulva spp.* when included in diets for simple-stomached animal species (a.o Cañedo-Castro et al., 2019; Øverland et al., 2019).

Besides beneficial attributes, challenges to include *U. laetevirens* in broiler diets are present such as a high mineral content (Biancarosa et al., 2017; Øverland et al., 2019) which can induce diarrhea (Koreleski et al., 2010) or lead to lower inclusion levels due to formulation constraints (maximum nutrient level). Furthermore, seaweeds including *Ulva* spp*.* are poorly digested by broilers (Bikker et al., 2020; Stokvis et al., 2022), leading to a low nutrient availability and poor performance. In conjunction with these nutritional challenges, the quantities of seaweed produced and processed are currently still relatively low leading to challenges regarding economic viability.

Potential solutions for the high mineral content can be washing using fresh water (Neveux et al., 2014) while the economic feasibility can be improved by implementing a biorefinery approach (a.o Bikker et al., 2016, 2020; Torres et al., 2019). By creating multiple fractions through biorefinery, valuable components can be extracted for use in the pharmaceutical, chemical or food industry (e.g., Holdt and Kraan, 2011). The then more cost-effective biorefined co-products are potential feed ingredients (Torres et al., 2019). This fractionation might, however, result in a feed ingredient of lower nutritional value compared to the original product. In the biorefinery concept, the high water content of seaweed facilitates fractionation by pressing, yielding a liquid fraction containing most soluble components including minerals, and a solid fraction containing mostly insoluble components including cell wall material. Additional treatment of the latter fraction by, for example, enzymes has been suggested (Stokvis et al. 2021a; Van Krimpen and Hendriks, 2019; Bikker et al., 2016) to be a potential strategy to improve nutrient availability for broilers.

However, in in a recent study (Matshogo et al., 2021), pre-treatment of seaweed meal with an exogenous fibrolytic enzyme mixture did not improve growth performance, a number of physiological parameters, and meat quality traits in broiler chickens. Recently in our laboratory using 5 (wk 1 and 2) and 10% (wk 3) *U. laetevirens* co-products in diets for broilers, a proteolytic enzyme treatment reduced nutrient digestibility and led to a higher FCR, whereas untreated *U. laetevirens* inclusion led to a lower FCR compared to a basal diet. The differences in FCR were only observed in the third (last) week of the trial (Stokvis et al., 2022). Furthermore, a reduced crypt dept and villus length in the duodenum, as well as a lower blood plasma interleukin-13 level were observed in birds fed the diet enriched with untreated vs. treated *U. laetevirens*. Intestinal histological characteristics like villus height and crypt depth can be used to assess gastrointestinal functioning and uptake capacity.

In the current study, it is hypothesized that the effects of seaweed product inclusion in diets are depending on the other dietary ingredients, and might thus exert a different effect in different diet types. The current study aimed to confirm the results of our previous trial, and to further investigate the effects of a proteolytic enzyme treatment of *U. laetevirens* co-product (fraction after washing and pressing) on digestibility and health-related parameters when included in a standard corn-soy diet and a diet based on protein sources derived from European countries.

## MATERIALS AND METHODS

The animal experiment was conducted at the facility of Wageningen University & Research in Wageningen, the Netherlands. All experimental procedures were approved by the Animal Care and Use Committee of Wageningen University & Research, the Netherlands (AVD40100202010104).

### Seaweed Harvesting and Processing

*Ulva laetevirens* was obtained from and processed by Olmix S.A. (Olmix Group, Bréhan, France) and harvested from the beach in France near Guisseny on September 30, 2014 and immediately washed with fresh water and then frozen until further processing. After thawing, *U. laetevirens* was ground to 50 to 1,000 nm particles (Inotec I175CDI-75D) and pressed twice using a belt press (Flottweg BFRU 800, Vilsbiburg, Germany) at 6 bar with intermediate rehydration (DM = 196 g/kg) using fresh water. The enzymatic treatment consisted of the addition of broad-spectrum endo-protease (0.5% Neutrase, Novozymes; 0.8 AU-N/g) to the *U. laetevirens* rehydrated cake (co-product) on a dry weight basis at 50°C for a duration of 5 h under low agitation, followed by a 10-min enzymatic inactivation step at 80°C. Both untreated (**U-**) and treated (**U+**) *U. laetevirens* co-products were air-dried at 60°C for 48 and 30 h, respectively followed by 60 h at 50°C up to 90% DM. Finally, all *U. laetevirens* products were ground to pass a 1-mm sieve before inclusion in the experimental diets. The composition and density of both *U. laetevirens* co-products are listed in Table 1.

**Table 1:** Analyzed nutrient content of the untreated and enzymatically^1^ treated seaweed (*Ulva laetevirens*) co-products.

ItemComponent	Untreated	Treated
Gross nutrient content (g/kg dry matter)		
Dry matter	896.0	888.0
Ash	272.3	274.8
Nitrogen (N)	21.4	22.3
Crude protein^2^	107.1	111.7
Crude fiber	97.1	83.3
Crude fat	5.6	6.8
Calcium	29.6	31.2
Phosphorous	1.3	1.3
Density (g/cm^3^)	0.692	0.653

1 Neutrase (Novozymes; 0.8 AU-N/g).

2 Calculated as N × 5.0 as per Angell et al. (2016).

### Animals and Housing

A total of 624 one-day-old male broilers (Ross 308, Morren, Lunteren, the Netherlands) with an average BW of 42.8 g were randomly assigned to one of 48 pens with 13 birds per pen whereafter lighter and heavier birds were exchanged between pens to ensure each pen was kept within a 3% difference of the average pen weight (556.8 g) with the starting pen weight for each taken to be 13 × 42.8 g. Each pen (1.85 × 1.10 m) had a solid floor covered with wood shavings. At arrival, all birds were vaccinated against infectious bronchitis and against Newcastle disease at d 15. Five days prior to the dissection of the birds of a pen (d 29, 30, or 31), bedding material and solid floors were replaced by slatted floors to enable excreta collection. Each pen was assigned to one of 6 treatments in a completely randomized block design with 8 replicate pens per treatment. Ambient temperature was maintained at 34°C for the first 2 d and, thereafter, gradually reduced to 20°C on d 27 and maintained at this temperature until the end of the experiment. A 23L:1D photoperiod was applied during the first 3 d, whereafter the dark period was increased by 1 h every day until a 16L:8D schedule was achieved. Birds had ad libitum access to feed and water. At the end of the experiment all birds were euthanized with an intracranial sodium pentobarbital injection before sample collection. Due to practical limitations related to the number of birds, and the number of measurements and samples taken per bird, euthanasia and dissection was performed per pen per replicate of treatments, either at d 30, 31 or 32.

### Experimental Diets

All starter (d 0–13) and grower (d 14-end of experiment) diets were formulated to meet or exceed requirements of all nutrients for broilers (CVB, 2019). The grower diet was supplemented with 5 g/kg titanium (**Ti**) dioxide and 1 g/kg cobalt-ethylenediamine tetra-acetic acid (**Co-EDTA**) as indigestible solid and liquid phase markers to allow determination of digestibility values. All diets were produced by Research Diet Services (Wijk bij Duurstede, the Netherlands), and fed as pellets (starter: 2.5 mm, grower: 3.2 mm). In total 6 diets were formulated, based on 2 diet types: a corn-soy based diet (**S**) and a diet based mainly on European protein sources (**EU**). For either diet type, a basal diet (**B**: **SB** and **EUB**) and two seaweed diets with (**SU+, EUU+**) or without (**SU-, EUU-**) the enzyme pre-treatment were formulated. The SU-, SU+, EUU- and EUU+ diets during d 0–6, d 7–13 and d 14-end of the experiment consisted of 100, 97.5, and 95% (w/w) basal diet with 0, 2.5, and 5% (w/w) U- or U+ seaweed, respectively. The ingredients of the diets and analyzed nutrient composition are presented in Tables 2 and 3, respectively. The water holding capacity (**WHC**; Table 3) of the diets was determined (AACC 2010) by soaking 1.0 ± 0.05 g feed pellets in deionized water in a 50 mL falcon tube for 60 min. After centrifugation (10 min at 4,000 × *g*), samples were left to drain for 15 min by placing the tubes at a 45° angle. The WHC was calculated as initial sample weight minus drained sample weight.

**Table 2:** Composition of the basal and untreated (−) and enzymatically^1^ treated (+) seaweed (*Ulva laetevirens*) co-product containing starter (d 0 to 13) and grower (d 14 to end of experiment) diets for broilers.

	Starter diet						Grower diet					
	Soy-based			European protein based			Soy-based			European protein based		
		*U. laetevirens*			*U. laetevirens*			*U. laetevirens*			*U. laetevirens*	
Ingredient (g/kg)	Basal^2^	-	+	Basal^2^	-	+	Basal	-	+	Basal	-	+
Corn	479.5	449.4	449.2	290.1	264.2	264.4	400.0	400.0	400.0	200.0	200.0	200.0
Wheat	150.0	150.0	150.0	200.0	200.0	200.0	299.0	232.1	232.6	315.7	250.6	251.1
Soybean meal	227.3	226.1	226.1	60.0	60.0	60.0	161.0	167.0	166.0	50.0	50.0	50.0
*U. laetevirens*-	-	25.0	-	-	25.0	-	-	50.0	-	-	50.0	-
*U. laetevirens+*	-	-	25.0	-	-	25.0	-	-	50.0	-	-	50.0
Rapeseed meal	-	-	-	50.0	45.0	45.0	-	-	-	50.0	50.0	50.0
Sunflower meal	80.8	80.4	80.4	80.0	80.0	80.0	75.0	75.0	75.0	80.0	80.0	80.0
Peas	-	-	-	160.0	160.0	160.0	-	-	-	160.0	160.0	160.0
Corn gluten feed	-	-	-	40.0	40.0	40.0	-	-	-	40.0	40.0	40.0
Potato starch	-	-	-	40.0	41.0	40.8	-	-	-	10.0	13.0	12.0
Palm fat (34.0 MJ)	-	-	-	-	-	-	-	-	-	24.0	33.5	33.5
Soybean oil (37.5 MJ)	16.5	26.0	26.3	35.0	43.0	43.0	17.0	35.0	35.0	24.0	33.5	33.5
Premix^3^ (5 g/kg)	5.0	5.0	5.0	5.0	5.0	5.0	5.0	5.0	5.0	5.0	5.0	5.0
Finely ground lime	13.5	11.8	11.7	14.2	12.5	12.3	10.8	7.1	7.0	11.1	7.4	7.2
Monocalcium phosphate	13.6	13.6	13.6	13.4	13.4	13.5	9.2	9.2	9.2	8.2	8.5	8.6
Salt	2.5	1.3	1.3	1.5	0.8	0.8	2.0	0.0	0.0	1.5	0.0	0.0
Sodium bicarbonate	1.8	1.8	1.8	2.6	2.0	2.0	2.4	1.9	1.9	2.6	1.4	1.4
L-Lysine HCl	4.2	4.2	4.2	3.5	3.5	3.5	5.1	4.8	4.9	4.6	4.3	4.5
DL-Methionine	2.8	2.8	2.8	2.7	2.7	2.7	2.7	2.7	2.8	2.7	2.7	2.8
L-Threonine	1.2	1.2	1.2	0.8	0.7	0.8	1.6	1.4	1.5	1.5	1.3	1.4
L-Valine	0.6	0.6	0.6	0.1	0.1	0.1	1.1	0.9	1.0	1.0	0.8	0.9
L-Arginine	0.7	0.8	0.8	1.1	1.1	1.1	1.6	1.5	1.6	1.4	1.3	1.4
L-Isoleucine	-	-	-	-	-	-	0.5	0.4	0.5	0.7	0.7	0.7
Titanium dioxide	-	-	-	-	-	-	5.0	5.0	5.0	5.0	5.0	5.0
Cobalt-EDTA	-	-	-	-	-	-	1.0	1.0	1.0	1.0	1.0	1.0
Total	1,000.0	1,000.0	1,000.0	1,000.0	1,000.0	1,000.0	1,000.0	1,000.0	1,000.0	1,000.0	1,000.0	1,000.0

1 Neutrase (Novozymes; 0.8 AU-N/g).

2 All birds were fed their respective basal diet from d 0 to 6.

3 Provided per kg of diet: vitamin A, 10,000 IU; vitamin D_3_, 2,500 IU; vitamin E, 50 mg; vitamin K_3_, 1.5 mg; vitamin B_1_, 2.0 mg; vitamin B_2_, 7.5 mg; vitamin B_6_, 3.5 mg, vitamin B_12_, 20 µg; niacin, 35 mg; D-pantothenic acid, 12 mg; folic acid, 1.0 mg; biotin, 0.2 mg; Fe, 80 mg; Cu, 12 mg; Mn, 85 mg; Zn, 60 mg; I, 0.8 mg; Se, 0.15 mg.

**Table 3:** Analyzed nutrient content of the basal and untreated (−) and enzymatically^1^ treated (+) seaweed (*Ulva laetevirens*) co-product containing starter (d 0–13) and grower (d 14-end of experiment) diets as fed to the broilers.

	Starter diet						Grower diet					
	Soy-based			European protein based			Soy-based			European protein based		
Item		*U. laetevirens*			*U. laetevirens*			*U. laetevirens*			*U. laetevirens*	
Component	Basal^2^	-	+	Basal^2^	-	+	Basal	-	+	Basal	-	+
Gross nutrient content (g/kg dry matter (DM)												
Dry matter (g/kg)	888.7	893.6	893.7	893.1	896.9	897.2	883.8	886.7	884.6	889.3	889.7	888.8
Ash	60.2	63.1	63.8	59.3	62.2	61.8	60.0	63.4	63.9	57.7	64.7	64.2
Nitrogen	36.6	37.1	35.9	35.7	36.4	36.1	33.5	34.0	34.4	33.8	33.8	33.2
Crude protein^3^	228.6	231.6	224.3	222.9	227.2	225.7	209.6	212.7	215.0	211.5	211.0	207.7
Neutral detergent fiber	91.9	102.2	102.3	127.3	130.1	127.1	100.9	116.3	111.4	126.7	138.9	138.7
Acid detergent fiber	40.7	44.8	43.9	56.2	57.2	57.3	42.9	49.2	49.7	58.5	66.3	65.7
Acid detergent lignin	1.4	4.1	3.4	7.7	9.3	8.8	4.2	4.5	6.0	8.3	8.2	9.3
Crude fat	47.2	54.5	55.8	65.5	72.0	73.7	43.6	56.3	54.9	79.5	94.6	95.2
Sugar	51.0	49.3	48.7	43.3	40.9	42.6	44.6	44.0	43.7	42.9	40.4	40.3
Starch	430.3	431.8	411.3	426.0	405.5	388.8	470.1	444.2	440.5	434.9	396.3	387.5
Calculated total fiber^4^	182.7	169.8	196.2	183.0	192.3	207.4	172.1	179.5	181.9	173.5	193.0	205.2
Macro minerals (g/kg DM)												
Calcium	-	-	-	-	-	-	8.9	7.9	7.7	8.6	8.2	7.8
Phosphorus	-	-	-	-	-	-	6.8	6.4	6.5	6.9	6.8	6.9
Potassium	-	-	-	-	-	-	8.8	9.0	9.1	8.2	8.4	8.4
Sodium	-	-	-	-	-	-	1.8	1.5	1.5	1.7	1.5	1.5
Chloride	-	-	-	-	-	-	3.3	2.1	2.3	3.0	2.4	2.4
Magnesium	-	-	-	-	-	-	2.1	3.0	3.0	2.2	3.0	3.0
Sulfur							2,359.1	4,076.8	3,967.9	2,934.8	4,428.7	4303.4
Micro minerals (mg/kg DM)												
Iron	-	-	-	-	-	-	195.2	326.5	331.8	211.4	348.5	361.7
Copper	-	-	-	-	-	-	17.5	22.0	24.9	20.8	19.7	21.4
Manganese	-	-	-	-	-	-	119.9	113.3	118.7	118.1	127.0	121.0
Zinc	-	-	-	-	-	-	105.2	102.1	105.7	110.8	102.9	106.9
Arsenic	-	-	-	-	-	-	0.1	0.4	0.3	0.1	0.3	0.3
Cadmium	-	-	-	-	-	-	0.2	0.2	0.2	0.2	0.17	0.2
Mercury	-	-	-	-	-	-	<0.01	<0.01	<0.01	<0.01	<0.01	<0.01
Lead	-	-	-	-	-	-	0.2	0.4	0.4	0.2	0.4	0.4
Nickel	-	-	-	-	-	-	2.4	2.9	2.8	2.6	3.0	3.0
Selenium	-	-	-	-	-	-	0.3	0.3	0.3	0.3	0.3	0.3
Calculated AME (MJ/kg)	12.06	12.04	12.06	12.37	12.33	12.34	12.33	12.28	12.29	12.59	12.56	12.57
Water holding capacity (g/g)	-	-	-	-	-	-	1.47	1.78	1.64	1.41	1.75	1.67

- not analyzed.

1 Neutrase (Novozymes; 0.8 AU-N/g).

2 All birds were fed their respective basal diet from d 0 to 6.

3 Calculated as N × 6.25.

4 Calculated as 1,000−ash−crude protein−crude fat−sugar−starch.

### Performance Measurements

Feed intake (**FI**) and water intake (**WI**) were recorded weekly per pen. Average BW per pen was determined upon arrival at the experimental facility, and again at d 7, 14, 21, and 28. The feed conversion ratio (**FCR**) was calculated as: total pen FI over the period/(Pen BW end of period–pen BW start of period + pen BW of dead or culled birds) with FI per bird corrected for mortality calculated as: FCR × BW gain.

### Sample Collection and Chemical Analyses

Excreta were collected qualitatively during two days before dissections, after which the birds of the corresponding pens were euthanized. Ileal contents were collected from the distal 40 cm of the ileum, anterior to the ileocecal junction of birds and pooled per pen. Excreta and ileal chyme were stored at −20°C until further processing. Before chemical analyses, excreta, and ileal chyme were freeze-dried, and all samples were ground to pass a 1-mm diameter screen. The U- and U+ were analyzed for DM (ISO 6496, 1999), ash, (ISO 5984, 2002), nitrogen (**N**; ISO 5983, 2005), crude fat (ISO 6492, 1999), crude fiber (ISO 6865, 2000), and Ca and P (ISO 27085, 2009), and their density (g/cm^3^) was determined. Starter diets were additionally analyzed for Na, K and Cl (ISO 27085, 2009; ISO 6495, 2015), starch (ISO 15914, 2004), sugar (EC 152, 2009), neutral detergent fiber (**NDF**; ISO 16472, 2006), acid detergent fiber (**ADF**), and acid detergent lignin (**ADL**; ISO 13906, 2008). Furthermore, grower diets were additionally analyzed for Fe, Mn, Mg, Zn, and Cu (ISO 27085, 2009) as well as As, Cd, Pb, Hg, Co, Se, Ni, and S (DIN EN 15763, 2009), in addition to Ti and Co. The markers were measured after ashing and microwave digestion using inductive coupled plasma optical emission spectrometry (**ICP-OES**). The ileal samples were analyzed for DM, ash, N, NDF, Ti, and Co, and the fecal samples were analyzed for DM, ash, N, crude fat, NDF, Ti, and Co. Uric acid was extracted using saturated lithium carbonate, and after centrifugation (3,000 rpm, 10 min) and subsequent dilution of 0.25 mL extract with 0.2 mL 0.2M hydrochloric acid and 4.55 mL demineralized water, it was measured using a uric acid kit (HUMAN Diagnostics) according to the manufacturer's instructions. Calculated total fiber was calculated as 1,000−ash−(N × 6.25)−crude fat−starch−sugar, and this fraction represents non-starch polysaccharides (**NSP**) + lignin.

### Health-Related Parameters

From 3 birds per pen with a BW close to the average pen BW, additional samples were collected. After euthanasia, the gizzard was separated from the proventriculus and the duodenum, and the full gizzard weighed. Gizzard contents were removed by rinsing with tap water and gently dried using a paper towel before the empty gizzard was weighed. From 2 of the same 3 birds per pen, the duodenum was separated from the gizzard and the jejunum before the pancreas was removed from the duodenal loop. A 1 cm piece of the proximal duodenum was dissected out just before the loop before the sample was gently rinsed in a physiological salt solution (0.9% NaCl) to remove remaining digesta before being stored in a phosphate buffered 10% formalin fixative at 4°C until further analyses. Before analyses, tissue samples were rinsed twice with tap water, and once with 70% alcohol, upon storage in 70% alcohol. The samples were cut in rings of ∼3 mm length, placed in histology cassettes and embedded in paraffin using the Leica TP1020 tissue processor (Leica Microsystems B.V., Amsterdam, the Netherlands). The embedded tissue samples were cut in 5-µm thin sections, stretched, and placed on glass slides. Samples were stained using Mayer's hematoxylin and eosin standard staining protocols. A Leica DM6b microscope and LASX software (Leica Microsystems B.V.) were used to measure villi length, crypt depth, tunica muscularis thickness and cross section of the duodenal lumen. From each sample, a maximum of 30 intact villi, 30 crypts, 6 cross sections, and 60 muscularis layer thickness were measured, of which the averages were taken as values per sample. Villus length was defined as the distance from the tip of a villus to the villus-crypt junction. Crypt depth was defined as the distance from the villus-crypt junction to the circular muscle layer. The tunica muscularis thickness was defined as the distance between the start of the circular muscle layer to the serosa. The cross section was defined as the maximum distance from the start of the circular muscle layer on opposite sides of the duodenum. The villi length to crypt depth ratio and the cross-section per kg BW were calculated.

### Calculations and Statistical Analyses

Performance parameters were calculated using FI and BW measurements over time. Apparent pre-cecal digestibility and apparent total tract digestibility of nutrients in the experimental diets were calculated, using Ti and Co as markers according to the following equation:

$$\text{DC}\left(X\right)=\left(1-\frac{\left[\text{Marker}\right]\text{diet}\;\times \left[X\right]\text{sample}}{\left[\text{Marker}\right]\text{sample}\;\times \left[X\right]\text{diet}}\right)\times \;100$$

where DC(X) is the apparent digestibility coefficient of nutrient X in % and [Marker]_diet_, [Marker]_sample_, [X]_diet_, and [X]_sample_ are the concentrations of the marker and nutrient X in the diet and digesta or excreta sample in g/kg, respectively. Apparent total tract nitrogen digestibility was calculated with fecal nitrogen corrected for nitrogen originating from urine with the use of uronic acid.

Data were analyzed using SAS statistical software (version 9.4, SAS Institute Inc., Cary, NC). For all data, a general linear model with contrast statements was used to determine 1) differences between birds fed the S diets and those fed the EU diets (SB, SU- and SU+ vs. EUB, EUU- and EUU+), 2) effect of seaweed inclusion per se (SB and EUB vs. SU-, SU+, EUU-, and EUU+), 3) effects of enzyme pre-treatment (SU- and EUU- vs. SU+ and EUU+) and 4) interaction effects between Diet type and seaweed inclusion per se, and diet type and enzyme pre-treatment. Model assumptions and goodness of fit were evaluated through normal distribution of residuals. Outliers identified by studentized residual >3 standard deviations from the sample mean were excluded from the analyses. Data are presented as means unless stated otherwise with differences among means with a probability <0.05 considered significant.

## RESULTS

Upon dissection, 3.5% of the birds originating from different pens and treatments were found to have ascites. No significant treatment effect was found on the incidence of ascites.

### Nutritional Composition

The enzymatically treated seaweed had a higher crude protein content and lower density compared to the untreated counterpart (Table 1). Due to isonitrogenous and isoenergetic diet formulation, S and EU diets differed in levels of fibrous components, crude fat, starch, and sugar (Table 3). The average WHC of U diets (1.71) was numerically higher than that of B diets (1.44) and the average WHC of U- diets (1.77) was higher than that of U+ diets (1.66). All analyzed microminerals in the diets were within the limits based on the European regulations for animal diets (EG 1334/2003; EC 32/2002), and were not majorly impacted by the enzymatic treatment.

### Diet Type Effect

In wk 1 and 2 of the experiment, a higher water intake (+27, and +63 mL per bird; *P* = 0.015 and *P* < 0.001, respectively) and water:feed (+0.17 and +0.09 mL/g; *P* = 0.013 and *P* = 0.016, respectively) were observed in birds fed S vs. EU diets (Table 4). In wk 3 and 4, birds fed S vs. EU diets had a higher BW (+41 and +64 g/bird; *P* < 0.001 for both), BWG (+33 and +23 g/bird; *P* < 0.001 and *P* = 0.008, respectively) and FI (+25 and +27 g/bird; *P* = 0.003 and *P* = 0.031, respectively), as well as a lower FCR in wk 3 (−0.05 g/g; *P* < 0.001). Based on the Co-EDTA marker, pre-cecal OM digestibility of S diets was higher than that of EU diets (*P* < 0.001; Table 5). Increased gizzard weight (empty: +1.3 g/kg BW and full: +4.7 g/kg BW) and contents (+3.7 g/kg BW) were observed in birds fed EU vs. S diets (*P* < 0.001 for all; Table 6). Furthermore, longer villi (+150 µm; *P* = 0.010) and increased (*P* = 0.0281) villus height:crypt depth were observed in birds fed EU vs. S diets.

**Table 4:** Effect of inclusion of 2.5 (d 7–13) and 5% (d 14-end of experiment) untreated (−) and enzymatically^1^ treated (+) seaweed (*Ulva laetevirens*) co-product in broiler diets (basal) based on soy or European protein sources on performance parameters.

	Soy-based diets (SD)			European protein-based diets (ED)								
Period		*U. laetevirens* (U)			*U. laetevirens*			*P*-values^3^				
Parameter^2^	Basal (B)^4^	-	+	Basal^4^	-	+	SEM	SD vs. ED (DT)	B vs. U	Enzyme (E)	DT × E	DT × U
D 0–7 (starter diet 1)												
Body weight gain (g)	167	166	168	166	163	170	1.4	0.793	-	-	-	-
Feed intake (g)	163	164	169	164	161	167	1.2	0.662	-	-	-	-
Feed conversion ratio (g/g)	0.97	0.98	1.00	0.99	0.98	0.99	0.003	0.951	-	-	-	-
Water intake (mL)	499	492	507	464	464	488	5.7	0.015	-	-	-	-
Water:feed (mL/g)	3.07	3.01	2.99	2.74	2.89	2.92	0.037	0.013	-	-	-	-
Body weight d 7	210	209	211	209	206	212	1.4	0.793	-	-	-	-
Mortality (% per pen)	0.0	0.0	3.8	1.9	1.0	0.0	0.4	0.690	-	-	-	-
D 7–14 (starter diet 2)												
Body weight gain	381	378	380	371	371	378	2.4	0.173	0.849	0.396	0.636	0.505
Feed intake	443	447	450	438	439	448	2.8	0.351	0.251	0.314	0.557	0.940
Feed conversion ratio	1.19	1.18	1.18	1.18	1.18	1.18	0.002	0.549	0.988	0.811	0.946	0.650
Water intake	933	944	955	849	891	904	10.4	<0.001	0.155	0.605	0.964	0.360
Water:feed	2.06	2.11	2.13	1.95	2.06	2.02	0.022	0.016	0.066	0.796	0.648	0.918
Body weight d 14	591	587	590	580	577	591	3.4	0.271	0.886	0.277	0.522	0.627
Mortality	0.0	1.0	3.0	1.0	0.0	1.0	0.4	0.455	0.434	0.235	0.675	0.198
D 14–21 (grower diet)												
Body weight gain	524	498	497	474	479	466	3.9	<0.001	0.077	0.458	0.216	0.033
Feed intake	747	739	736	702	732	714	4.3	0.003	0.564	0.267	0.451	0.064
Feed conversion ratio	1.43	1.47	1.48	1.48	1.53	1.53	0.006	<0.001	<0.001	0.909	0.979	0.904
Water intake	1,344	1,410	1,385	1,284	1,400	1,392	12.9	0.416	0.002	0.578	0.792	0.229
Water:feed	1.80	1.91	1.88	1.83	1.91	1.95	0.014	0.242	<0.001	0.857	0.355	0.888
Body weight d 21	1,114	1,091	1,087	1,054	1,057	1,057	5.9	<0.001	0.351	0.867	0.847	0.167
Mortality	1.9	4.9	2.1	0.0	1.9	1.0	0.6	0.072	0.195	0.226	0.524	0.970
D 21–28 (grower diet)												
Body weight gain	702	680	679	671	670	652	4.5	0.008	0.071	0.310	0.341	0.477
Feed intake	10,31	1,058	1,034	1,000	1,030	1,012	6.6	0.031	0.191	0.157	0.845	0.795
Feed conversion ratio	1.47	1.54	1.52	1.49	1.54	1.55	0.007	0.125	<0.001	0.732	0.398	0.987
Water intake	1,813	1,904	1,961	1,780	1,927	1,910	16.6	0.514	<0.001	0.435	0.151	0.717
Water:feed	1.76	1.80	1.90	1.78	1.87	1.89	0.013	0.294	<0.001	0.029	0.116	0.895
Body weight d 28	1,816	1,771	1,766	1,726	1,726	1,709	9.0	<0.001	0.134	0.538	0.703	0.185
Mortality	2.9	3.1	4.2	5.0	0.0	1.9	0.7	0.439	0.274	0.312	0.795	0.108

- not applicable.

1 Neutrase (Novozymes, 0.8 AU-N/g).

2 Each value is based on 8 replicate pens of 13 birds.

3 Statistical contrasts: Diet type (SD basal, SD *U. laetevirens*- and SD *U. laetevirens*+ diets) vs. (ED basal, ED *U. laetevirens*- and ED *U. laetevirens*+ diets), Basal vs. *U. laetevirens*: (SD basal and ED basal diets) vs. (SD *U. laetevirens*-, SD *U. laetevirens*+, ED *U. laetevirens*- and ED *U. laetevirens*+ diets), Enzyme: (SD *U. laetevirens*- and ED *U. laetevirens*- diets) vs. (SD *U. laetevirens*+ and ED *U. laetevirens*+ diets).

4 All birds were fed their respective basal diet from d0-6.

**Table 5:** Effects of inclusion of 2.5 (d 7–13) and 5% (d 14-end of experiment) untreated (−) and enzymatically^1^ treated (+) seaweed (*Ulva laetevirens*) co-product in a broiler grower diet (basal) based on soy or European protein sources on apparent pre-cecal and total tract nutrient digestibility in broilers based on the titanium dioxide (Ti) and cobalt-ethylenediamine tetraacetic acid (Co-EDTA) markers.

	Soy-based diets (SD)			European protein-based diets (ED)								
Digestibility^2^		*U. laetevirens* (U)			*U. laetevirens*			*P*-values^3^				
Nutrient	Basal (B)	-	+	Basal	-	+	SEM	SD vs. ED (DT)	B vs. U	Enzyme (E)	DT × E	DT × U
Apparent pre-cecal (%) based on Ti												
Ash	44.9	58.2	58.6	40.7	29.0	29.8	1.78	<0.001	0.865	0.948	0.835	<0.001
Organic matter	74.4	82.0	81.8	70.0	65.2	66.1	1.01	<0.001	0.473	0.916	0.347	<0.001
Nitrogen	80.7	86.3	85.8	79.5	77.1	77.5	0.60	<0.001	0.241	0.976	0.515	<0.001
Apparent total tract (%) based on Ti												
Ash	32.2	27.8	42.0	32.1	24.0	40.8	0.96	0.386	0.474	<0.001	0.009	0.597
Nitrogen	77.8	75.3	74.1	74.9	71.9	71.3	0.37	<0.001	<0.001	0.245	0.551	0.852
Crude fat	68.1	56.9	48.1	51.4	22.5	21.2	2.63	<0.001	<0.001	0.227	0.008	<0.001
Neutral detergent fiber	6.3	19.6	16.4	17.1	20.3	20.1	0.87	0.003	<0.001	0.239	0.193	<0.001
Apparent pre-cecal (%) based on Co-EDTA												
Ash	29.7	17.8	19.7	29.0	19.6	19.9	0.93	0.814	<0.001	0.382	0.553	0.437
Organic matter	67.3	64.6	64.0	64.2	60.6	61.3	0.50	<0.001	<0.001	0.950	0.455	0.856
Nitrogen	75.4	72.9	71.8	75.5	74.0	74.3	0.51	0.140	0.012	0.690	0.479	0.300
Apparent total tract (%) based on Co-EDTA												
Ash	20.1	15.1	33.3	23.2	15.6	35.0	1.20	0.455	0.216	<0.001	0.253	0.719
Nitrogen	73.9	70.9	70.3	71.4	68.8	68.5	0.33	<0.001	<0.001	0.437	0.701	0.477
Crude fat	62.5	49.4	40.4	44.6	13.9	15.3	2.66	<0.001	<0.001	0.370	<0.001	<0.001
Neutral detergent fiber	-10.4	5.4	3.9	5.4	11.5	12.3	1.27	<0.001	<0.001	0.865	0.371	0.001

1 Neutrase (Novozymes, 0.8 AU-N/g).

2 Each value is based on 8 replicate pens of 13 birds.

3 Statistical contrasts: Diet type (SD basal, SD *U. laetevirens*- and SD *U. laetevirens*+ diets) vs. (ED basal, ED *U. laetevirens*- and ED *U. laetevirens*+ diets), Basal vs. *U. laetevirens*: (SD basal and ED basal diets) vs. (SD *U. laetevirens*-*,* SD *U. laetevirens*+*,* ED *U. laetevirens*- and ED *U. laetevirens*+ diets), Enzyme: (SD *U. laetevirens*- and ED *U. laetevirens*- diets) vs. (SD *U. laetevirens*+ and ED *U. laetevirens*+ diets).

**Table 6:** Effects of inclusion of 2.5 (d 7–13) and 5% (d 14-end of experiment) untreated (−) or enzymatically^1^ treated (+) seaweed (*Ulva laetevirens*) co-products in a broiler diet (basal) based on soy or European protein sources (Diet type) on gastrointestinal tract characteristics.

	Soy-based diets (SD)			European protein-based diets (ED)								
Tissue		*U. laetevirens* (U)			*U. laetevirens*			*P*-values^4^				
Parameter	Basal (B)	-	+	Basal	-	+	SEM	SD vs. ED (DT)	B vs. U	Enzyme (E)	DT × E	DT × U
Gizzard (g/kg BW)^2^												
Gizzard weight empty	9.8	9.4	9.8	10.8	11.3	10.8	0.15	<0.001	0.895	0.784	0.190	0.507
Gizzard weight full	14.5	13.2	13.1	18.2	18.3	18.5	0.46	<0.001	0.552	0.997	0.871	0.360
Gizzard content	4.6	3.8	3.7	7.4	8.0	7.8	0.39	<0.001	0.867	0.932	0.977	0.400
Duodenum (µm)^3^												
Villus length (VL)	1,909	1,873	1,758	1,967	2,050	1,972	0.029	0.010	0.658	0.222	0.789	0.252
Crypt depth (CD)	121	120	128	121	124	120	0.002	0.796	0.667	0.745	0.373	0.891
VL:CD	16.2	14.8	14.2	16.4	16.9	17.0	0.410	0.0281	0.482	0.838	0.737	0.183
Muscularis thickness	123	129	126	129	124	122	0.002	0.755	0.886	0.747	0.902	0.343
Cross section (µm/kg BW)	3,121	3,364	3,237	3,184	3,467	3182	0.051	0.565	0.033	0.021	0.308	0.852

1 Neutrase (Novozymes, 0.8 AU-N/g).

2 Each value is based on 6 replicate pens of 3 birds.

3 Each value in the table is based on 6 replicate pens of 2 birds.

4 Statistical contrasts: Diet type (SD basal, SD *U. laetevirens*- and SD *U. laetevirens*+ diets) vs. (ED basal, ED *U. laetevirens*- and ED *U. laetevirens*+ diets), Basal vs. *U. laetevirens*: (SD basal and ED basal diets) vs. (SD *U. laetevirens*-*,* SD *U. laetevirens*+*,* ED *U. laetevirens*- and ED *U. laetevirens*+ diets), Enzyme: (SD *U. laetevirens*- and ED *U. laetevirens*- diets) vs. (SD *U. laetevirens*+ and ED *U. laetevirens*+ diets).

### Ulva Laetevirens *Co-Product Inclusion Effect*

Dietary inclusion of 5% U in wk 3 and 4 increased FCR (+0.05 and +0.06 g/g; *P* < 0.001 for both), water intake (+83 and +129 mL/bird; *P* = 0.002 and *P* < 0.001, respectively), and water:feed (+0.10 and +0.10 mL/g; *P* < 0.001 for both). Furthermore, the apparent pre-cecal ash (*P* < 0.001), OM (*P* < 0.001), and N (*P* = 0.012) digestibility coefficients based on the Co-EDTA marker were all lower after U inclusion. The duodenal cross section was larger in birds fed U vs. B diets (+160 µm/kg BW; *P* = 0.033).

### Enzyme Treatment Effect

Water:feed in the last wk of the experiment was higher for birds fed U- vs. U+ diets (+0.06 mL/g; *P* = 0.029), as were apparent total tract, but not pre-cecal ash digestibility based on both the Ti and Co-EDTA markers (both *P* < 0.001). The duodenal cross section was larger in U- vs. U+ diets (+206 µm/kg BW; *P* = 0.021).

### *Diet Type ×* Ulva Laetevirens *Inclusion Effect*

Dietary U inclusion in wk 3 led to a lower BWG in birds fed S (−27 g/bird) vs. EU diets (−1.5 g/bird; Diet type × U effect; *P* = 0.033). For birds fed S diets, U led to higher apparent pre-cecal digestibility of all nutrients based on the Ti marker, whereas for birds fed EU diets a lower digestibility was observed after U inclusion (*P* < 0.001 for all nutrients). Based on both the Ti and Co-EDTA markers, a stronger reduction in apparent total tract crude fat digestibility was observed after U inclusion in EU vs. S diets (Ti and Co-EDTA *P* < 0.001) as well as a stronger increase in apparent total tract NDF digestibility after U inclusion in S vs. EU diets (Ti *P* < 0.001; Co-EDTA *P* = 0.001).

### Diet Type × Enzyme Treatment Effect

No Diet type × Enzyme effects were observed for performance. Apparent total tract crude fat digestibility was further reduced when U+ was added to EU diets (−30% absolute) than when added to S diets (−16% absolute) based on both the Ti (*P* = 0.008) and Co-EDTA markers (*P* < 0.001).

### Ti vs. Co-EDTA Marker

Generally lower digestibility values were observed based on the Co-EDTA vs. the Ti marker (Table 5). The apparent total tract digestibility coefficients were lower for ash (−28%), N (−4.8%), crude fat (−16%), and NDF (−72%) calculated with the Co-EDTA marker.

## DISCUSSION

### Diet Type Effect

The observed higher water intake in wk 1 and 2 of birds fed the S vs. EU diets is not in line with observations by Jiménez-Moreno et al. (2016). These authors reported an increased water intake in birds fed higher insoluble NSP levels at young ages. In the current study, dietary levels of NDF and ADF were twice that of the levels of Jiménez-Moreno et al. (2016) although here we did not differentiate between soluble and insoluble fibers or NSP. Solubility of fibrous components strongly determines their biological effects (Mateos et al., 2013). A larger WHC of a diet also increases water intake (Jiménez-Moreno et al., 2009). However, WHC in our study was similar for EU vs. S diets, hence not explaining the high water intake of birds fed S diets.

Despite diet formulation aimed at similar quantities of digestible amino acids and calculated AME (based on ingredient values), small differences between diets were observed, such as a lower AME:apparent digestible protein ratio of the S (66.64–72.87 MJ/kg) vs. EU (74.86–78.15 MJ/kg) diets. Contrary to our findings, in the literature feed intake, weight gain and feed efficiency (in g BW gain /kg feed) of male broilers from 0 to 3 wk of age improved when birds were fed higher AME:digestible protein ratios (70.4 vs. 77.7 MJ/kg; Gous et al., 2018), although the absolute AME in our diets (12.28–12.59) correspond with the AME of their 70.4 MJ/kg digestible protein diet (AME: 12.3 MJ/kg). This indicates that the EU diets, containing the higher AME:digestible protein ratio, were similar in energy but only higher in protein compared to the S diets. When Gous et al. (2018) investigated body composition, protein gain was not increased with increasing AME:digestible protein ratio diets, whereas they observed an increased lipid gain, meaning energy was not the limiting factor, whereas that might have been the case for the birds in our study.

Another cause for a reduced feed intake can be in response to a combination of fiber sources (sugar beet pulp and rice hulls; Sadeghi et al., 2015) and in particular of soluble fibers (Rahmatnejad and Saki, 2016). Since the EU diet contained a range of fiber sources, this could also explain the observed lower feed intake. In addition, according to Annison (1993), higher soluble NSP levels of for example wheat, increase digesta viscosity, reduce the diffusion rate of digestive enzymes into digesta, hamper their interaction at the mucosal surface and hence reduce nutrient utilization. In the current experiment, indeed a reduced digestibility of the EU vs. S diet and an increased FCR of birds fed those diets was observed, although wheat inclusion in the EU vs. S diets was only slightly higher.

The EU diets contained 24 to 35 g/kg palm fat, whereas the S diets did not. Valencia et al. (1993) reported no differences in growth and efficiency due to oil source, but observed an increased BW of 21-day-old broilers fed diets with increasing levels of oil from 0 to 2 and 4%, but a decrease with further increasing oil levels to 6, 8, and 10%, while maintaining constant energy levels. In our study, the EU diets contained 4.8 to 6.7% soybean oil+palm fat, and compared to the 1.7 to 3.5% in the S diets, this might also have had a negative effect on performance.

Heavier gizzards (+13%) and gizzard contents (+35%) were observed in birds fed the EU vs. S diets, which is in accordance with data in the literature. For example, 33% heavier gizzards were observed as a result of higher dietary fiber levels (ground to <2.5 mm; Jiménez-Moreno et al., 2009, 2019). Additionally, studies of Jiménez-Moreno et al. (2010, 2019) observed a reduced gizzard pH and increased apparent total tract retention of N in broilers of 21 d of age in response to increasing dietary fiber levels. Surprisingly, we observed the opposite: a lower apparent total tract N digestibility in the EU vs. S diets. This could be explained by differences in the level of all dietary fiber components. For example, NDF in our diets ranged from 101 to 139 g/kg DM, whereas 84 g/kg DM was the highest level in the study of Jiménez-Moreno et al. (2019). Potentially the optimal level of fibers had been surpassed and the surplus of fibers in the EU diets had a negative effect on nutrient digestibility. Moreover, different responses to different fiber sources are observed in various studies in the literature (Jiménez-Moreno et al., 2010).

Longer villi correspond to a higher nutrient uptake capacity (Cañedo-Castro et al., 2019). The longer villi in birds fed the EU vs. S diets was contrary to our hypothesis, as we expected a decrease in villus length in response to more abrasive digesta and sloughing off of cells due to higher fiber inclusion. The lack of differences in crypt depth in birds fed the EU vs. S diets indicates that the turnover rate of enterocytes was not different between birds fed the S and EU diets. Similar observations are reported for crypt depth in response to increasing fiber levels in the literature (Tejeda and Kim, 2020). Furthermore, longer villi are reported in response to diets higher in fiber (Tejeda and Kim, 2020; Rahmatnejad and Saki, 2016). The latter studies report that the longer villi are due to more stimulus of abrasive insoluble fibers, whereas soluble fibers seem to decrease villi height. This would indicate that in the EU diet contained more abrasive insoluble fibers compared to the S diet. This was in line with our research setup including higher fiber levels in the EU diet, although in this study no differentiation was made between soluble and insoluble fibers. Despite the observed histomorphological differences between treatments, no differences were observed in nutrient absorption, or in performance between birds fed the EU and S diets.

### Ulva Laetevirens *Inclusion*

Macro- and micromineral levels were all within the limits for safe use in broiler diets, although the ash content of the *U. laetevirens* co-products (270 g/kg DM) will likely cause problems when these are included at higher levels than in the current experiment.

Despite the differences in ash, fiber, crude fat and starch contents in the U vs. B diets, U inclusion at 2.5% did not affect performance parameters of the broilers in wk 2. The higher water intake and water:feed observed in birds fed the U vs. B diets in wk 3 and 4, with dietary U inclusion levels of 5%, are corresponding to the higher mineral content and the higher WHC of the U diets. A high water intake could lead to diarrhea, suboptimal bird health, and reduced performance (Guiry and Blunden, 1991; Koreleski et al., 2010). The higher water intake in birds fed the U diets might have induced the negative effect on FCR by flushing out nutrients, although diarrhea was not visually observed. The higher WHC observed for the U diets might have caused more bulky feed/chyme boluses due to the greater water retention at a similar feed intake. In this study, this is reflected by a wider duodenum (calculated per kg BW) in birds fed the U vs. B diets, but not by a larger gizzard. This was also not reflected in a lower feed intake due to a fuller crop with the high water intake due to minerals and high WHC of the diets.

In the S diets, U inclusion led to an increased apparent pre-cecal digestibility of all nutrients, but to a decreased apparent total tract nutrient digestibility, and BWG was decreased in wk 3. This increase in apparent pre-cecal nutrient digestibility in birds fed the seaweed supplemented S diets might be a beneficial effect of the addition of fibers, originating from the seaweed products, in the diet. In contrast, U inclusion in the EU diets led to a decrease in apparent pre-cecal ash, OM and N digestibility, and some small changes in digestibility of the fibrous dietary components, without affecting BWG. Potentially, there was already sufficient fibrous material present in the EUB diets to optimize digestion, and the maximum degradation capacity might have already been reached, meaning the extra fibrous material merely hampered digestion capacity. Moreover, changes in the physicochemical conditions in the gastrointestinal tract, due to the different diet types, may have resulted in a variation in responses in nutrient digestibility and performance traits (Tejeda and Kim, 2020; Choct et al., 2010).

### Enzyme Treatment Effect

As a consequence of the addition of N in the form of enzymes as part of the enzymatic treatment, the N content was slightly higher (+4.2%) in U+ vs. U- products, although due to the relatively low enzyme inclusion levels the dietary N content was only marginally higher in the U+ vs. the U- diets. In relation to nutrient digestibility, the enzyme treatment only affected apparent total tract ash digestibility, which was increased in the U+ vs. U- diets, and decreased apparent total tract crude fat digestibility in the S but not in the EU diets. The decreased WHC of U+ vs. U- diets did not result in differences in for example water intake or gizzard content, contrary to data in the literature (Jiménez-Moreno et al., 2009). The general lack of observed differences indicates that the enzyme treatment did not improve the nutritional value of the seaweed products for broilers. If the enzyme treatment did release protein, peptides or amino acids, this might have been subject to complex forming, for example, with heavy metals (Ashmead, 1992), and this may have hampered nutrient digestibility.

### Digestibility Based on Ti and Co-EDTA Markers

Generally, the recovery of Ti (Jagger et al., 1992; Sales and Janssens, 2003; Kavanagh et al., 2001) is higher than that of Co (Marais, 2000; De Vries et al., 2014; Udén et al., 1980) in various gastrointestinal segments, consequently leading to higher calculated digestibility coefficients (De Vries and Gerrits, 2018). Digestibility coefficients in our study were indeed higher when calculated based on Ti. A high correlation was observed in digestibility coefficients based on the Ti and Co markers, especially for apparent total tract nutrient digestibility, with correlation coefficients ranging between 0.932 and 0.997 (*P* < 0.001 for all). It is known that Co(II)EDTA is not completely stable, which might lead to absorption of Co in the gastrointestinal tract which violates the assumption of inertness of the markers (De Vries and Gerrits, 2018). Furthermore, the liquid phase of digesta, in which the Co-EDTA marker is present, passes through the gastrointestinal tract at a different rate compared to the solid phase containing the Ti marker, and might accumulate in parts of the gastrointestinal tract as illustrated by De Vries et al. (2014). Reflux, in particular of the insoluble digesta fraction, might for example increase the concentration of the solid phase marker (Ti) in the ileum (Sacranie et al., 2012). This might lead to higher calculated pre-cecal compared to total tract nutrient digestibility values. This was indeed observed in the present study, although only for the high fiber S diets (SU- and SU+) but not for the EU diets. One notable difference is that of the apparent pre-cecal ash and N digestibility calculated using the 2 markers, specifically in the S diets. Calculated based on Ti, both ash and N digestibility increase with U inclusion, whereas based on Co they decrease with U inclusion. This might be related to the fiber fraction that holds the N and increases the reflux of solid phase digesta, whereas the Co follows the liquid digesta phase and increases in concentration in the ceca as mentioned before (Sacranie et al., 2012). The higher apparent pre-caecal vs. total tract N digestibility is in line with data in the literature for fiber-containing ingredients (Moughan et al., 2014). It is likely a consequence of a net synthesis of AAs from nitrogenous compounds (e.g., uric acid) by microbiota residing in the ceca and colon, which are subsequently recovered in the excreta. Our experimental diets, containing undigestible NDF, appear to have simulated N fixation and bacterial growth.

Digestibility of NDF was low in all experimental groups, as is expected for fibers in broilers and poultry in general. Some negative NDF digestibility coefficients were observed based on calculations using the Co marker. This indicates that either the Co marker is partly taken up in the gastrointestinal tract as discussed earlier in this section, or the Co marker, that follows the soluble or liquid phase of the digesta is not following the insoluble fiber. Hence, the Co marker appears to be less suitable compared to the Ti marker for calculating digestibility of insoluble fibrous fractions in broilers. Neutral detergent fiber was determined by a washing method designed for feed materials employing neutral detergent, and gravimetrically measured (ISO 16472, 2006). Bacteria present in the digesta can adhere to the dietary NDF and are not completely washed off the fiber during this procedure, thereby, increasing the weight of the NDF fraction recovered in the excreta. The latter consequently leads to a lower apparent total tract digestibility coefficient (De Jonge et al., 2015). Fiber is poorly digestible and fermented by poultry, and combined with the overestimation of NDF in the excreta, this can explain negative NDF digestibility values.

## CONCLUSIONS

This study confirms the high mineral content of *U. laetevirens* and its relatively poor nutrient digestibility in broilers. Dietary *U. laetevirens* increased apparent pre-cecal digestibility of nutrients when included in a corn-soy based diet, but decreased apparent pre-cecal digestibility when included in an EU diet, although in both diet types *U. laetevirens* reduced rather than improved performance. The proteolytic enzyme treatment of an *U. laetevirens* co-product did not affect performance, nor did it increase nutrient digestibility, and is thus not suitable to increase the nutritional value of this seaweed co-product for broilers. No effects were observed on performance, gizzard development or histomorphological parameters, indicating that bioactive properties related to these measurements of the seaweed co-product were lacking.
